# Botany, Genetics and Ethnobotany: A Crossed Investigation on the Elusive Tapir's Diet in French Guiana

**DOI:** 10.1371/journal.pone.0025850

**Published:** 2011-10-03

**Authors:** Fabrice Hibert, Daniel Sabatier, Judith Andrivot, Caroline Scotti-Saintagne, Sophie Gonzalez, Marie-Françoise Prévost, Pierre Grenand, Jérome Chave, Henri Caron, Cécile Richard-Hansen

**Affiliations:** 1 Direction Etudes et Recherches Guyane, Office National de la Chasse et de la Faune Sauvage, Kourou, French Guiana, France; 2 Herbier de Guyane, UMR AMAP, IRD, Cayenne, French Guiana, France; 3 UMR AMAP, IRD, Montpellier, France; 4 UMR 0745 EcoFoG, INRA, Kourou, French Guiana, France; 5 UPS 3188 IRD, OHM Oyapock du CNRS, Cayenne, French Guiana, France; 6 Laboratoire Evolution et Diversité Biologique, UMR 5174 CNRS, Université Paul Sabatier, Toulouse, France; 7 INRA, Université de Bordeaux, UMR1202 BIOGECO, Cestas, France; University of Western Ontario, Canada

## Abstract

While the populations of large herbivores are being depleted in many tropical rainforests, the importance of their trophic role in the ecological functioning and biodiversity of these ecosystems is still not well evaluated. This is due to the outstanding plant diversity that they feed upon and the inherent difficulties involved in observing their elusive behaviour. Classically, the diet of elusive tropical herbivores is studied through the observation of browsing signs and macroscopic analysis of faeces or stomach contents. In this study, we illustrate that the original coupling of classic methods with genetic and ethnobotanical approaches yields information both about the diet diversity, the foraging modalities and the potential impact on vegetation of the largest terrestrial mammal of Amazonia, the lowland tapir. The study was conducted in the Guianan shield, where the ecology of tapirs has been less investigated. We identified 92 new species, 51 new genera and 13 new families of plants eaten by tapirs. We discuss the relative contribution of our different approaches, notably the contribution of genetic barcoding, used for the first time to investigate the diet of a large tropical mammal, and how local traditional ecological knowledge is accredited and valuable for research on the ecology of elusive animals.

## Introduction

The lowland tapir (*Tapirus terrestris* Linnaeus, 1758) is the largest terrestrial herbivore of the Neotropics and it has been present in the rainforest vegetation since before the Pleistocene [1]. There is strong evidence that the large tropical rainforest fauna is being depleted in many parts of the world due to forest fragmentation and/or unsustainable game hunting [2], [3], and the tapirs are especially threatened in the Neotropics [4], [5]. However, the ecological consequences of this decline of large herbivores remain poorly documented [6]–[9]. In particular, this decline may lead to shifts in ecological function [6]–[9] and in plant species diversity [10]–[16]. This topic has motivated a lot of recent research (*e.g.* [4], [17]–[21]), but there still is a dire need for more quantitative knowledge about the tapirs diet so as to clarify their ecological function role in the neotropical forest ecosystems.

The lowland tapir is an herbivorous species, known to consume a wide variety of plants, but its diet is not fully understood [22]. One reason is that there may be regional variation in its diet. Indeed, the diversity in plant species consumed and the degree of frugivory of the lowland tapir's diet are suspected to vary regionally and seasonally, most probably as a consequence of resource availability. A second reason is that the tapirs are elusive animals, and most estimates of their diet must rely on indirect methods. The classic approach to studying tapir's diet involves observing browsing signs (*e.g*. [23]), identifying the diet in macroscopic and histological analyses of food residuals in faeces (*e.g.* [24]) or analyzing the stomachs of killed animals (*e.g.* [25]). All these methods are tedious, and they typically rely on incomplete samples, possibly underestimating quantitatively the resource used by tapirs and revealing its diet only in part.

A second promising approach to understand the diet of tapirs is the genetic-based identification of tissue fragments directly from faeces (*e.g.* [26], [27]) through the DNA barcoding technique [28]. Species identification is based on a small sequence (*ca* 500 nucleotides) that may be targeted with a single primer set for all organisms (*e.g.* [29]). It is essentially an improvement of the previous techniques, in that it enables a quicker and more reliable identification of plant tissue, especially when it has been degraded in the animal's digestive tract.

A third source of knowledge on the ecology of large game species is based on traditional ecological knowledge (*e.g.* [30]–[32]). Traditional knowledge can be defined as the knowledge that indigenous people capitalise on due to observations and transmission over very long periods of time [33]. This traditional ecological knowledge has recently called attention for biodiversity assessments [34] and natural resources management [35], but these developments are frequently out-of-sight to mainstream biologists [36], [37]. It would be important to assess whether this traditional ecological knowledge does shed a new light on the ecological niche of the lowland tapir, and only a cross-comparison of the various approaches can yield such an assessment.

Here we assess which food items are used by lowland tapirs in the Guianan shield, and we contrast methods for their identification. The ecology of the lowland tapir has been much less investigated in the Guianas than in Brazil or Peru (e.*g.* [22], [38]–[40], but see [41]). This region also presents fewer flooded forests, fewer and smaller patches of palm trees and no natural salt licks, all factors known to affect the ecology of lowland tapirs, at least in Peru [42]–[44]. The different ecological environment in the Guianas may imply the tapir's diet to be slightly different there as well. In this paper, we illustrate how the original combination of (1) classic and (2) molecular approaches and (3) traditional ecological knowledge can help understand the diet of the lowland tapir in French Guiana.

## Methods

### Study area

**Figure 1:**
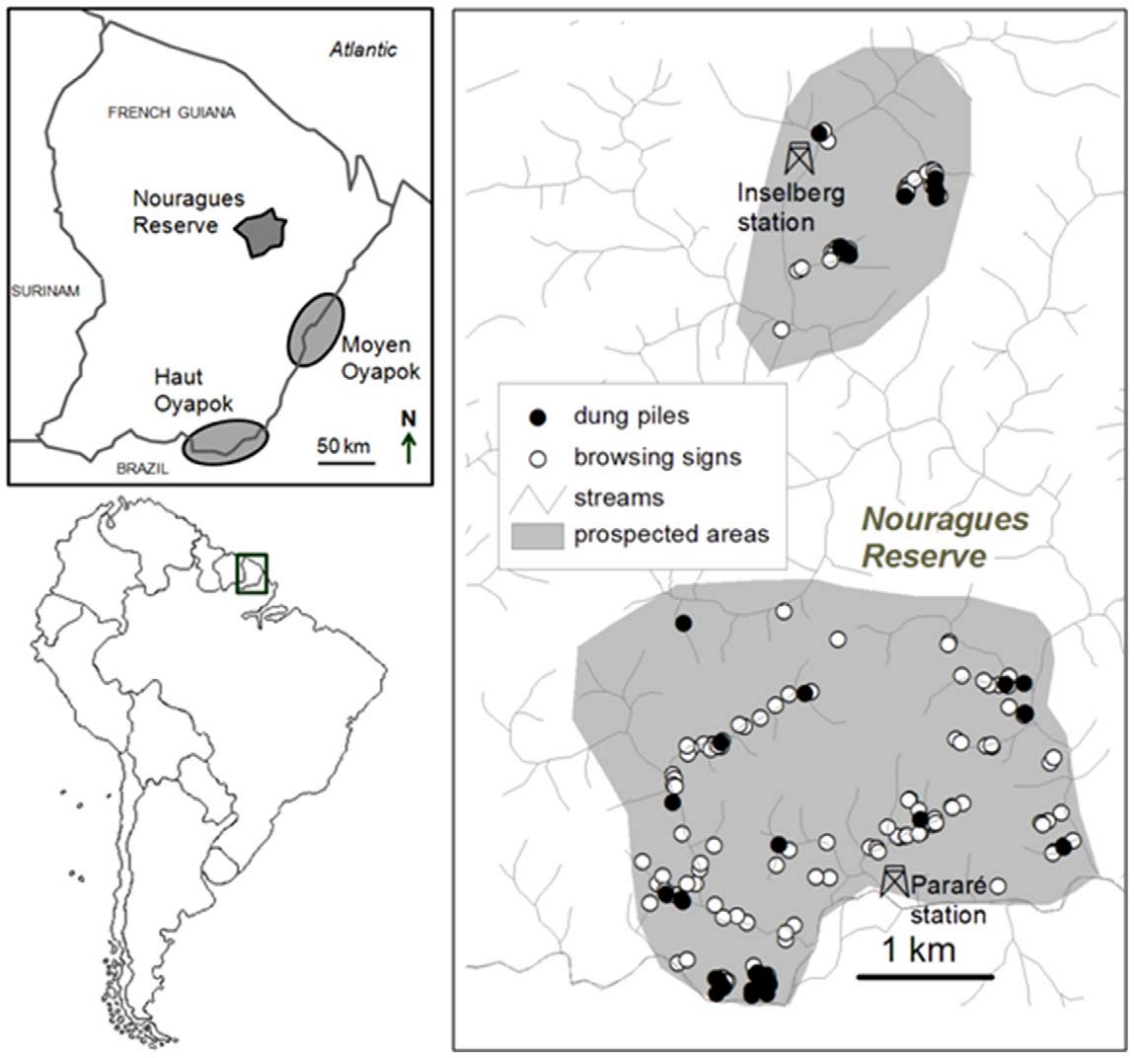
Location of the study areas and sites where tapir browsing signs and dung piles were collected.

The Nouragues Reserve (4°05′N, 52°40′W) is a 1000 km^2^ protected area in French Guiana, in the northern part of the Amazon rainforest (Fig. 1). It is characterized by a succession of small hills, 60–120 m asl, covered by an evergreen primary rainforest [45] (see also www.nouragues.cnrs.fr/). Annual rainfall averages 2880 mm, with a distinct dry season from September to November (<100 mm per month), and a shorter drier period around February and March. The local flora includes over 1700 angiosperm species [46]. Tree fruiting peaks in March-May and is minimal in August-September [47], [48].

Aside from the lowland tapir, the reserve shelters three other ungulate species: the red brocket deer (*Mazama americana* Erxleben, 1777), the grey brocket deer (*M. gouazoubira* Fischer, 1814), and the collared peccary (*Pecari tajacu* Linnaeus, 1758). The nomadic white-lipped peccary (*Tayassu pecari* Link, 1795) was once present but has not been sighted in over ten years.

### Botanical study

From January to July 2009, we prospected two areas in the Nouragues Reserve (Fig. 1). Whenever we crossed fresh tracks of tapirs, we carefully followed them to spot plants exhibiting distinct signs of recent browsing such as freshly broken stems, cut leaves and remaining petioles. We only recorded browsing signs with fresh tapir footprints underneath to minimize error due to counting signs from other ungulates. For each browsed plant encountered, a dried botanical voucher was prepared for further identification by expert botanists at Herbier de Guyane (CAY). These same botanic experts categorised the abundance of the browsed plants found in this study as uncommon, common, locally very common or very common in the areas of the surveys. These broad categories were based on the results of comprehensive floristic inventories in the Nouragues Reserve of both the trees, on two sample plots of 6 ha and 8 ha respectively [49], and of the understorey plants on 334 20-m^2^ (5 m×4 m) plots and on 400 25-m^2^ (5 m×5 m) plots, totalising more than 33000 understorey plant individuals inventoried.

### Faeces collection and sample preparation

Fresh tapir dung piles were collected at different locations (Fig. 1) from December 2007 to July 2009, with most of them collected after January 2009, through a systematic survey along the reserve streams. The consistent but irregular presence of dung piles in some sites suggested temporary latrine use. Greyish to blackish, odourless dung piles were judged old and were not sampled.

The dungs were washed and filtered through sieves of 1 mm mesh sizes. The particles were sorted in two categories: fruit (seed, pulp, fibrous pericarp) and fibre (stem, leaf). The fruit residuals were dried in a plant drying oven and stored with silica gel for further macroscopic identification using herbarium and photographed specimens as reference collections. From the fibre material, we sampled 410 homogenous and still green particles of about 1 cm^2^ that were stored in individual paper envelopes in plastic bags with silica gel for further genetic analyses.

### Genetic analysis of plant residuals

Plant DNA was extracted from the dung fibre samples using the Invisorb® Spin Plant Mini kit following the manufacturer protocol (Invitek GmbH, D13125, Berlin, Germany). PCR amplifications followed Shaw *et al.* [50]: the primers were *trnHGUG* [51] and *psbA* [52]. The chloroplastic DNA intergenic spacer region *trnH*-*psbA* [53] is known to be an interesting barcode to identify plants and rainforest plants in particular (e.g. [54], [55], [56] but see [57]). PCR products were cleaned using EXOSAP-IT (USB, Cleveland, Ohio). Sequencing reactions were performed using ABI Big Dye version 3.1 terminators and cycle-sequencing protocols. Sequencing reactions were purified with Sephadex ® G-10 from SIGMA-ALDRICH and loaded on an ABI 3130 XL capillary sequencer (Applied Biosystems, Foster City, CA). Sequences were aligned and edited using CodonCode Aligner version 3.0.1 (Codoncode Corporation, Dedham, MA). As recommended by Kress *et al.* [53], we kept the sequences longer than 120 base pairs.

The sequences were then matched against the sequences of identified plant species from the reference genetic database, using the blast algorithm as described in Gonzalez *et al.* [55]. The trnH-psbA database has been developed by Gonzalez *et al.* [55] for plants of the Nouragues reserve, and it was expanded for the purpose of the present study. It includes only plants of French Guiana and notably trees from the Nouragues reserve. The taxonomic resolution varies across groups: in some clades, the evolutionary history of the genus did not permit to reach lineage-sorting reducing the efficiency of genetic barcodes in the species discrimination [58]. In other clades, one can confidently assign a sequence to a species. In both cases, the queried sequence may be identified by using the alignment mismatch (calculated as the sum of the number of opening gaps with the number of mismatches divided by the alignment length) with known plant sequences. According to preliminary tests with sequences of the reference database, mismatches under 0.3%, 2.0% and 4% with best matching sequences reliably indicated same species, genus and family respectively. We used these criteria to identify the minimum number of best candidate taxa found in tapir dungs. The sequences are deposited on GenBank (see Table S1 for the accession numbers).

### Ethnobotanical data

Original information about plants known by native Indians to be eaten by lowland tapirs was collected from prominent elderly sages and hunters during two series of enquiries in Wayãpi villages of the Haut-Oyapock (Trois-Sauts) and of the Moyen-Oyapock (Camopi) regions (Fig. 1). Wayãpi are Tupi-Guarani people, who migrated from the Amazon River at the beginning of the eighteenth century. They have been isolated for one and a half centuries and have retained until today an astonishing knowledge about plants and animals [59]. The first ethnobotanical study was carried out between 1972 and 1976, with complements in 1989 and 1992 [60], [61]; the second, between 1980 and 1982, with complements in 1995 and 1996 (F. Renoux, unpubl. data). The ethnobotanists recorded information through open discussions while participating in everyday activities of the village, without any pre-set limit on the time for discussions or the topics that had to be covered. These surveys resulted in detailed information about the ecology of wildlife and, among others, a list of plant species known by Wayãpi to be eaten by lowland tapirs. The ethnobotanists identified these plants from direct observation of specimens pointed out by the Wayãpi and comparison of the sampled specimens to reference vouchers.

All necessary permits were obtained for the described field studies. The research program and the biologic material collection were approved and validated by the Conseil Scientifique Régional du Patrimoine Naturel (CSRPN) , the official authority for the national reserves. The program was also validated by the Conseil Scientifique du CNRS for the studies in the area of the reserve dedicated to the scientific research.

### Diet variety analysis

To evaluate the completeness of the species list produced by both classic and genetic approaches, we generated species rarefaction curves using 500 randomizations [62] with EstimateS Version 8.2 [63]. Total species richness was estimated by the Chao2 estimator [64]. Accumulated richness of fruit residuals in dung was calculated in function of the number of dung samples. Conversely, using browsing signs, that of browsed species was calculated in function of the number of prospected feeding sites. We attributed to a same feeding site the browsed plants observed along the same tapir track. In both cases, indeterminate species corresponding to unique taxa were also included. We assumed that unidentified DNA sequences from dung that differed by more than 5% belonged to different species (see [55]). To generate groups of sequences differing more than 5%, with 95% of their length compared, we used Blastclust [65]. Then, we estimated the minimum cumulated richness of plant species in the dung in function of the number of dung fibre samples sequenced, randomly sampled across the dungs.

## Results

### Diversity and variation of plants in the tapir's diet

**Figure 2:**
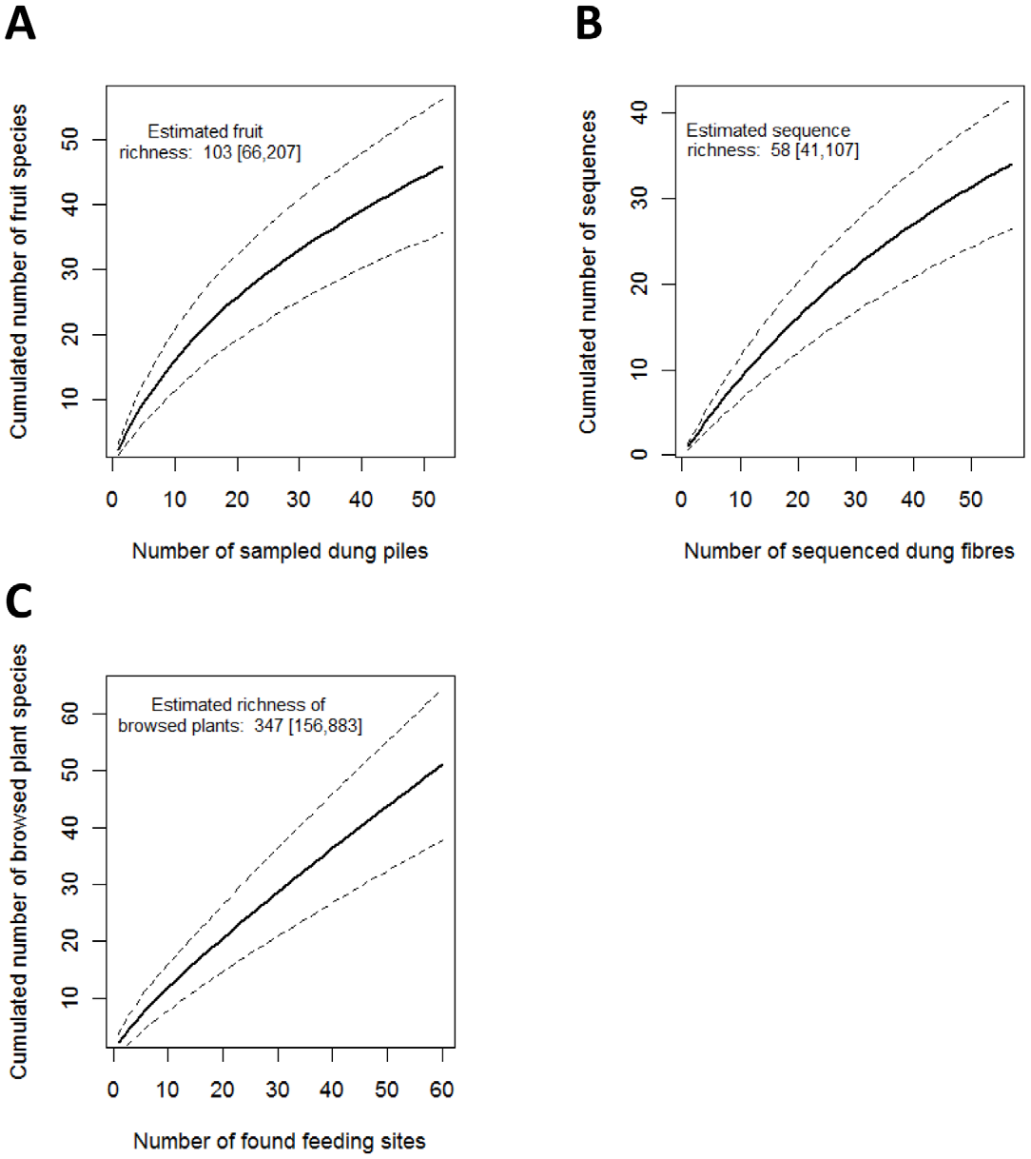
Species accumulation curves for plants eaten by tapirs in French Guiana from different sampling approaches. a) Cumulated richness of fruit residuals found in tapir dung samples, b) Cumulated richness of DNA sequences of individual fibres of browsed plants from 19 tapir dung piles, differing more than 5%, c) Cumulated richness of browsed plant species found in browsing signs. Total species richness is estimated by Chao2 estimators, with 95% confidence intervals given in square brackets.

Overall, we recorded 112 plant species in 98 genera and 50 families eaten by lowland tapirs either as fruits (42 plus at least 16 unidentified species, 41 genera, 13 families), as leaves (70 plus at least 13 unidentified species, 53 genera, 24 families) or as both (13 families, 5 genera) (Tables S2 and S3). The Rubiaceae, Melastomataceae and Sapotaceae families showed the greatest numbers of eaten species. All of the species accumulation curves from the botanical and genetic approaches were far from reaching an asymptote, indicating that an increased sampling effort would probably reveal much more species (Fig.2). One third of the identified browsed plants were tree species, another third shrub species, one quarter herbs and the rest lianas. Most of these were common (51%) and very common (29%) in the surveyed areas according to the local expertise (Table S2). We found more uncommon species in browsed shrubs (25%) and lianas (100%) than in trees (15%) and herbs (0%).

### Comparison of the approaches

Four families of browsed plants: the Melastomataceae, Rubiaceae, Cyclanthaceae and Sapindaceae and three genera: *Miconia* (Melastomataceae), *Psychotria* (Rubiaceae) and *Asplundia* (Cyclanthaceae) were systematically found by all three of our approaches. The taxa commonly found by any two of our approaches are indicated in Tables S2 and S3.

**Figure 3:**
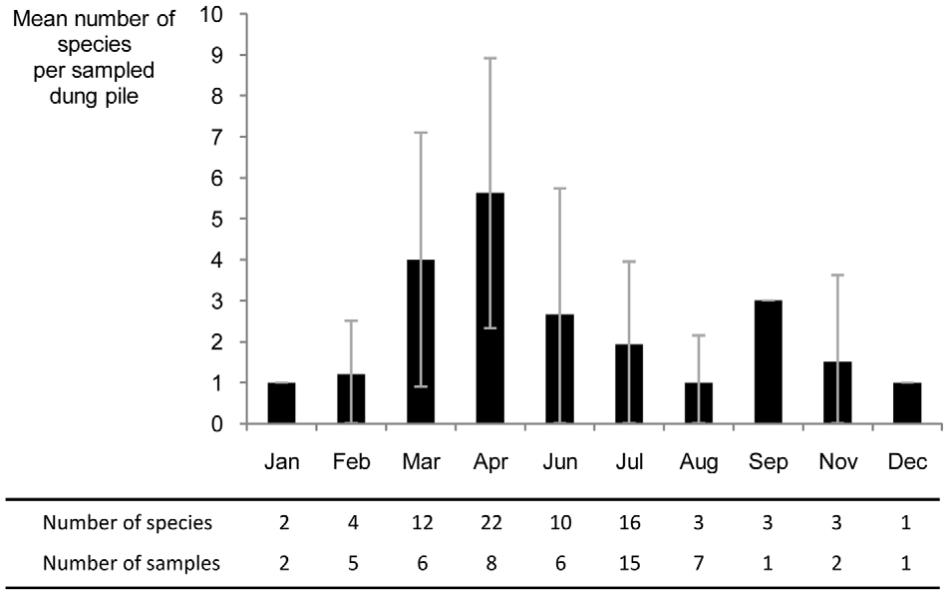
Seasonality of fruit variety in tapir dung. Error bars represent the standard deviations of the numbers of fruit species found in dung piles each month.

The botanical analysis of 73 browsing signs and of fruit residuals in 53 dung piles led to the identification of most of the species eaten as leaves or fruits (67% and 65% respectively). According to the Chao2 estimated richness, tapirs are expected to consume up to 103 fruits and browse 347 species (Fig. 2). The dung analysis further showed a seasonal change in the diversity of fruit residuals, with a major peak in April and a minor one in September-November (Fig. 3). This approach also indicated that for 69% of the fruit species found in the dung samples, the seeds were sometimes intact, and for 46%, they were always intact (Table S3) and could be dispersed by tapirs. Browsing signs were informative about the modalities of foraging by tapirs. Most of the time, tapirs had taken terminal leaves and twigs of tree seedlings and saplings, herbs and shrubs that they had sometimes broken down. Some plants also presented signs of previous browsing. Tapirs had browsed less selectively (i.e. most parts of the plants eaten) plants growing in tree-fall gaps and epiphytes on fallen logs (*e.g. Evodianthus funifer*, *Philodendron* spp.). Plant species browsed in tree-fall gaps made up 29% of the browsing signs.

We were able to amplify the DNA of 95 (23%) tapir dung fibre samples. Of these, we were able to successfully sequence 57 (14%) samples at the *trnH*-*psbA* locus (*i.e.* a sequencing success of 60%). Eleven samples could be confidently assigned to a species, 32 to a genus and 6 only to a family of the reference database. In total, 17 distinct families, 18 genera and 5 species were identified (Table S2). Compared to direct botanical identification, DNA barcoding enabled us to list 16 and 17 percent of new families and genera of browsed plants, respectively.

**Figure 4:**
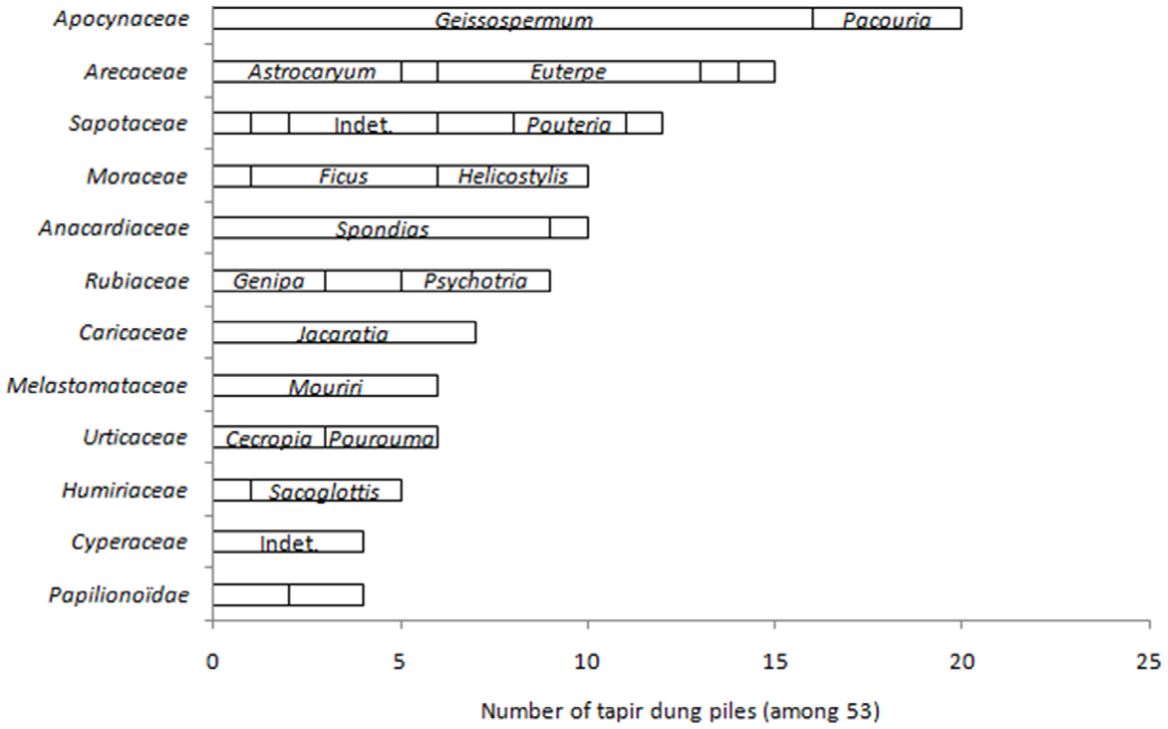
Frequency of most recurrent fruit taxa found in tapir dung piles from the Nouragues Reserve. We indicated families and genera found in more than four and three dung piles respectively. The length of each segment is proportional to the frequency of the considered genus.

The third approach used traditional ecological knowledge. Our ethnobotanical survey reported 19 and 26 species eaten by tapirs as fruits or leaves, respectively (Tables S2 and S3). From these, 14 and 22 species made up 33 and 31% of additional species eaten for fruits and leaves, respectively, compared to our other approaches. However, a large proportion of the families (70 and 71% of fruit and browse respectively) and genera (50 and 50% of fruit and browse respectively) in the indications of the Wayãpi indians were also listed in our direct analyses (Tables S2 and S3). These fruits were also among the most frequently found ones in the tapir dung piles (Fig. 4).

## Discussion

### New insights in tapir consumed taxa

**Figure 5:**
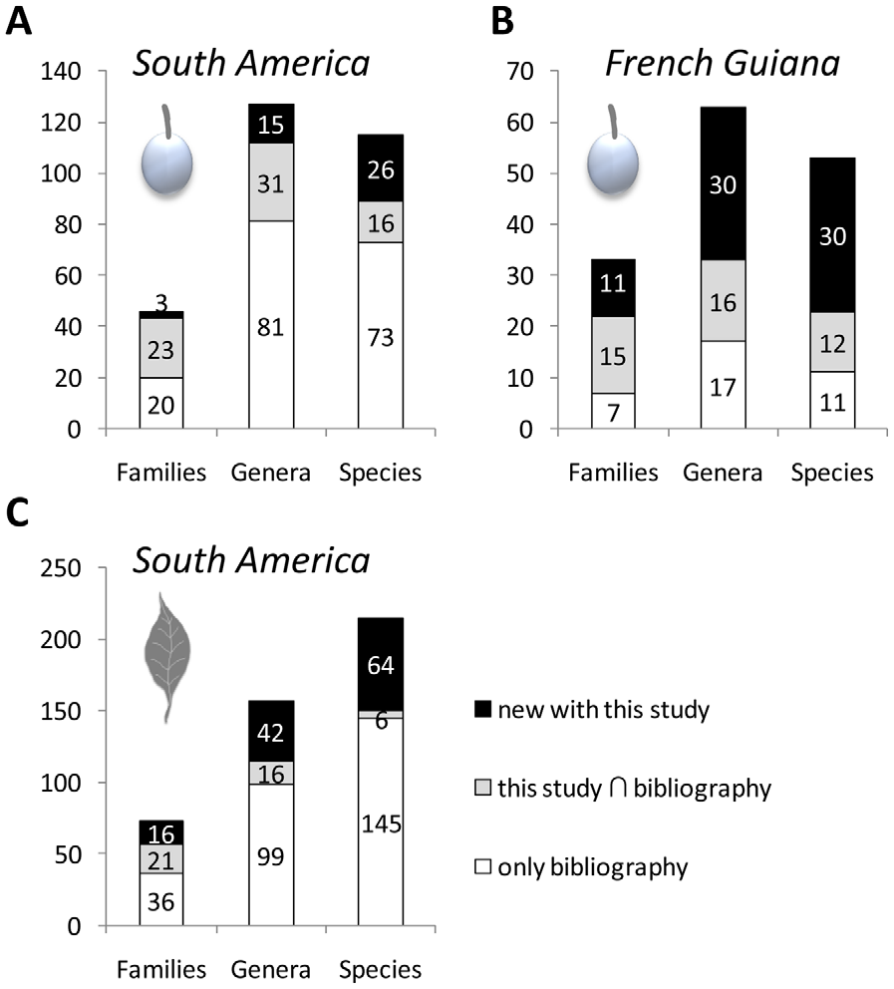
Numbers of identified plant taxa eaten by lowland tapirs, comparison with the bibliography. a) Comparison with the number of fruit taxa listed in South America. b) Comparison with the number of identified fruit taxa listed in French Guiana. c) Comparison with the number of identified browsed plant taxa listed in South America. Compared bibliography: Peruvian Amazon [22], [25], [42], [43], [83], North-Eastern Argentina [84], Bolivia [85], Venezuela [23], French Guiana [41] (list from their Table1), Brazil Amazon [38], [39], South-Eastern Brazil [6], [86]-[90].

Since the diet of lowland tapirs is diverse and the knowledge of consumed plants is limited by the amount of sampled materials [22], any new study is likely to discover new plant taxa consumed by tapirs. In the present study, we took a multidisciplinary approach and unveiled new species browsed by lowland tapirs (Table S4). This is unsurprising since the leaf diet received much less attention from the scientists (Fig. 5). We also extended the list of fruits eaten by lowland tapirs in French Guiana (see [41]) to 53 species, 63 genera and 33 families with 30 identified new species, 30 new genera and 11 new families (Fig. 5).

The variety of browsed plants reported in the present study (83 species: 70 identified plus 13 unidentified) is similar to that recorded by Salas and Fuller [23] in Venezuela, and by Montenegro [43] in Peru (both 88 species) but for different sampling procedures and efforts. Because our sampling effort was limited, we expect to find far more species browsed by tapirs in French Guiana.

The fruit variety found in the dungs was higher than that reported in Salas and Fuller [23] and Fragoso and Huffman [39] in Brazil when corrected for sample size. It was, however, far less than that predicted (46 *vs.* ∼78 for a similar dung sampling effort) by Tobler *et al.* [22] in Peru. It was also relatively less, when corrected for sample size, than that reported by Henry *et al.* [41] in lowland tapir stomach samples in French Guiana. Only three species mentioned by Henry *et al.* [41] are not listed in the Nouragues Reserve but the fruit diet differences (Fig. 5) may reflect differential spatial and temporal availability of fruits [48].

### Commonly eaten plants and regional differences in tapir diet

The low overlap of our browsed plant list with species recorded in the bibliography is striking (Fig. 5). It is certainly due to regional differences in the resources available to tapirs. Indeed, 73 and 44 percent of the species listed in the literature but not found in our study, have never been observed in the Nouragues Reserve or in French Guiana, respectively [66]. The high diversity and endemicity of the Guiana Shield's biota has been notably attributed to its varied topology [66]. Lowland tapirs may also compensate the absence of salt licks in the Guiana Shield by extending their diet to alternative plant species to fulfil their mineral requirements.

Interestingly, however, several genera of browsed plants detected in our study were commonly listed by other studies of lowland tapir's diet [23], [43], [67]. These were the understorey shrubs *Miconia* (Melastomataceae), *Psychotria* and *Faramea* (Rubiaceae), the epiphytic forb *Philodendron* (Araceae) and the forbs *Asplundia* and *Evodianthus* (Cyclanthaceae).

The fruits most frequently found in tapir dung piles of the Nouragues Reserve were also among those most often reported by other studies (listed in Fig. 5) in South America. These were the large juicy and fragrant fruits of *Spondias mombin*, *Helicostylis tomentosa*, *Ficus* spp., and *Bagassa guianensis*. However, we did not find the often cited palm-fruits *Mauritia flexuosa* and *Syagrus romanzoffiana* because both are absent from our study sites. Some other fruits frequently found in the dung piles we analysed had only been listed in French Guiana [41]: *Geissospermum laeve*, *Jacaratia spinosa*, *Mouriri collocarpa*, *Pacouria guianensis*, *Sacoglottis cydonioïdes* and these may be more specific of the lowland tapir's diet in the Guiana Shield.

### Does the diversity of tapir diet only reflect the diversity of available plants?

The recurrence of the above mentioned taxa suggests that they are important resources for lowland tapirs. Nevertheless, it is difficult to conclude about any foraging selectivity in lowland tapir without a systematic measurement of plant and fruit availability and use by tapirs in the environment, as undertaken by Salas and Fuller [23] and Tobler *et al.* [22]. Hence, the presence of uncommon shrub and liana species in the diet does not necessarily imply that they were selected. Likewise, the observed seasonal variation in the variety of consumed fruit seems to reflect their availability [47], [48] as well as opportunistic foraging (but see [42]), as also shown by Tobler *et al.* [22]. The most frequent fruit residuals in dung piles were among the ones found for longer periods of time or found at periods when more dung piles were sampled (*e.g. Jacaratia spinosa* and many *Sapotacae*) (Fig. 5; Table S3).

Nevertheless, tapirs obviously browsed selectively on plant parts. They clipped mainly the terminal and most recent parts certainly because these were more nutritive and less fibrous in spite of the possible presence toxins [68], [69]. Interestingly, the Amerindians interviewed also pointed this out. By selectively eating only small amounts of material from many different plant species, tapirs might also use several different detoxification pathways and eventually ingest larger amounts of food, as shown in other herbivores [68], [70]. Hence, the selectivity of plant parts by tapirs would be driven by their need to diversify their diet.

In contrast, tapirs appeared less delicate when consuming plants in tree-fall gaps. Gap plants, unlike plants living in habitats where light is limiting, have higher rates of leaf turnover, and invest less in leaf defence [71]. They also have significantly lower tannin leaf concentration, and lower toughness and fiber contents [72]. Hence, they constitute appetent resources for herbivores such as tapirs [23].

### Indices of impact of tapir foraging on vegetation

From most observed browsed signs, it appeared that plants could cope with regular but limited pruning by tapirs. Nevertheless, browsing signs could obviously be only detected in plants that survived the browsing. To quantify the actual impact predation of large herbivore can have on rainforest seedlings, exclosure experiments have been used [14]. To date, however, only a few such experiments have been published (*e.g.* [15]) and, to our knowledge, none of them have evaluated the specific impact of tapirs.

The dispersal role of tapir has been recently suspected in many other plant species than palm-trees (e.g. [22], [39]). We found that the seeds of 69% of species found in tapir dungs could be rejected intact, but in all cases in water although some dung piles were previously found in *terra firme* areas of the reserve. Forthcoming studies *in situ* should investigate whether these species can germinate after stream draining in the beginning of the dry season or be further dispersed by other animals.

If *Ficus*, *Cecropia*, *Bagassa*, *Euterpe* and many others are well known to be dispersed by a diverse guild of frugivores [73]-[75], *Geissospermum leave*, *Mouriri collocarpa*, *Pacouria guianensis* and *Sacoglottis cydonioides* produce particularly attractive, often juicy, fibrous and fragrant large fruits with resistant seeds whose dispersal could more specifically rely on tapirs or a few number of terrestrial frugivores. In our area, *G. leave* and *P. guianensis* were also eaten by red brocket deer according to Gayot *et al.* [76].

In contrast, we suspect cases of "passive frugivory", *i.e.* when the plant is foraged for vegetative parts but some small fruits are ingested by the same occasion, in a *Poaceae* and several understorey *Psychotria* (*Rubiaceae*). The seeds of some other fruits were systematically cracked (*e.g. Astrocaryum paramaca*) as observed in peccaries [16], suggesting that these seeds themselves represent resources for tapirs.

### Limits and complementarity of the approaches

The classic approaches of sampling (browsing signs and dung analysis) provided most of quantitative results reported in this study and most of the reported plant taxa. To date, observation of browsing signs has been the main approach to investigate the browse part of tapirs diet (*e.g.* [23], [43]). It enables a direct collection of the plants and informs about the modalities and environmental conditions of browsing by tapirs. However, since we searched for browsing signs along tapir tracks which are more easily found on soft soils, our plant list from browsing signs might be biased towards humid area species. This bias could be overcome by searching browsed signs along tapir trajectories revealed by telemetry studies. Another limitation is that too much damaged plants do not allowed us to collect a workable voucher for identification. Molecular identification by DNA barcoding could complement this approach. We showed here, for the first time, that the DNA of digested plant material found in lowland tapir dung can also be sequenced for identification. The low taxonomic redundancy of the sampled particles from dungs confirmed that tapir diet is very diverse. However, the expected richness of plants in dungs based on DNA barcoding alone was six times less than expected from classical approaches. One explanation is that only large particles have been sequenced and these may be less different from each other than would be any randomly chosen particles, leading to a biased estimation of variety. This difference may also be explained by the low similarity threshold we used to discriminate the sequences. The expansion of the reference genetic database to more species in French Guiana, especially local understorey herbaceous plants, lianas, and epiphytes, should allow to better estimate the diversity of tapirs diet thanks to a refined identification of consumed plants. Thus, at the present stage, we suggest that DNA barcoding approaches are most useful as a complement of classical approaches, and they are unlikely to supersede them.

Some other limitations of this method should be emphasized. The sequencing success appeared limited (14% of the treated plant samples) despite a costly procedure. In some plant families, the higher content of secondary metabolites may limit the success of extraction or amplification [77]. Also, DNA may be degraded by the condition of the samples, which limits the efficiency of the sequencing of long strands of DNA. One promising approach is to use shorter DNA barcodes, less likely to be degraded. Also new technologies such as next-generation sequencing could help retrieve far more information from the dung samples [78]. Increasing the capacity of sample treatment with high-throughput sequencing would both refine the information about the diversity and improve quantitative estimates of the diet (*e.g.* [79] but see [80]). We would notably expect to identify eaten fruit species whose seeds were spat before ingestion or too damaged to be identified, complementing the macroscopic analysis of fruit residuals in dungs. We hope to return to this question in a forthcoming contribution.

The ethnobotanical survey contributed to the identification of 10 and 19 new species eaten as fruits and browse respectively (comparison with the references given in Fig. 5). The ability of the Wayãpi people to name some lowland tapir food resources to the species illustrated a refined knowledge of their environment that is "not just about immediate technical solutions to everyday problems" [81], as also shown by Grenand [59]. Huntington *et al.* [30] stressed that there is no simple test for evaluating the reliability of information derived from traditional ecological knowledge. Nevertheless, the knowledge of the Wayãpi people is backed by the large overlap of the plant taxa with those found by other approaches in our study, and by other studies of tapir diet (common taxa: 88 percent of the families, 61 percent of the genera and 36 percent of the species) despite the outstanding plant species richness in the area. Although twice as many plants eaten for browse were indicated as for fruits, Amerindian hunters pay more attention to fruit foraging than browsing habits of tapirs (P. Grenand, pers. obs.). This suggests that the given list of browsed plants is biased towards species in which browsing is more easily observed. For instance, the Wayãpi name *‘tapi'i ka'a*’ for *Asplundia*, where large leaf browsing is particularly evident, means ‘tapir plant’. In contrast, the indicated fruits would rather correspond to species of trees under which the Wayãpi hunters are more likely to find various animal prey species (P. Grenand, pers. obs.). This knowledge could be useful to formulate hypotheses about the items eaten by other large mammals, such as deer, whose spoors are usually less easily detectable than for tapirs. The extension of such an approach to other local communities with a rich traditional ecological knowledge would certainly be also valuable. For example, the vernacular names of *Psychotria mapourioides* and *Sacoglottis cydonioïdes,* in Galibi (from which Aublet [82] derived the genus *Mapouria*) and Aluku languages respectively, both mean ‘tapir tree’.

This work benefited from the extended knowledge capitalised in French Guiana thanks to original local long-term botanical and ethno-botanical field studies as well the development of a local reference genetic database for plants. We acknowledge that applying this approach in other sites where less information is available is certainly not easy. Nevertheless, the development of the international genetic reference database, including tropical plants from more and more different sites, is promising for the identification of botanical samples. Our results also stress the importance of undertaking further field studies in botany, ethnobotany and animal-ecology and associating scientists from these different disciplines.

In conclusion, we confirmed important regional variations in the diversity and composition of the diet of lowland tapirs and found new plants used by these animals in north-western Amazonia. If tapirs alimentary plasticity is undeniable (see also [67]), the degree to which they are resilient to drastic changes in the floristic composition of their environment has still to be measured. We also confirmed the value of classic non-invasive approaches to study the diet of elusive herbivores. However, we stress that coupling these approaches with new telemetry and next-generation genetic methods, should refine the knowledge of the modalities and impact of their foraging behaviour, both at the population and individual scales. Finally, this research demonstrates that traditional ecological knowledge also provides a valuable source of ecological information to develop new research hypotheses on the ecology of elusive wildlife. We join other authors (e.g. [36], [37], [90]) to encourage integrative studies like this one, combining modern approach and traditional knowledge, to generate baseline ecological data for the better understanding of the ecosystems functioning and their management.

## Supporting Information

Table S1 **List of the sequenced samples with taxonomic identification and Genbank accession number.** Five sequences were not deposited in Genbank because they were less than 200 bp.
(DOCX)

Table S2 **List of plants browsed by tapirs.** Symbols are as follows: W: species communicated by the Wayãpi indians in the ethnobotanic surveys, B: species found by the botanical study , G: species found by genetic analyses. The abundance of these plant species in the surveyed areas was categorised by the botanist experts as uncommon (UC), common (C), locally very common (LVC) and very common (VC). "DOS" and "indet." stand for "depends on the species" and "indeterminate", respectively. For plants identified only at the family level, the abundance was given for the family. The names of the taxa found by more than one method are in bold and underlined.
(DOCX)

Table S3 **List of fruits eaten by tapirs.** Symbols are as follows: W: species communicated by the Wayãpi indians and D: species identified from the macroscopic analysis of fruit remains in dung. For the latter, we also indicated whether the seeds were intact (int.) or damaged (dam.) and the month of collection. The fructification periods observed during the ethnobotanical survey are indicated with *.The names of the taxa found by several approaches are in bold and underlined. Indeterminate species are indicated by "indet.".
(DOCX)

Table S4 **List of plants eaten by lowland tapirs in South America.**
(DOCX)
