# Supplementary material for: Botany, Genetics and Ethnobotany: A Crossed Investigation on the Elusive Tapir's Diet in French Guiana
Source: PLoS One. 2011 Oct 3;6(10):e25850. doi: 10.1371/journal.pone.0025850 (PMC3185057; doi:10.1371/journal.pone.0025850)
Supplement: Table S1 — List of the sequenced samples with taxonomic identification and Genbank accession number. Five sequences were not deposited in Genbank because they were less than 200 bp. (DOCX) [file pone.0025850.s001.docx]

**Supporting Information Table S1. List of the sequenced samples with taxonomic identification and Genbank accession number.** Five sequences were not deposited in Genbank because they were less than 200 bp.

|  |  |  |  |
| --- | --- | --- | --- |
| **Sequence** | **Genbank accession number** | **Identification** | **Taxonomic level** |
|  |  |  |  |
|  |  |  |  |
| 1 | BankIt1479670 seq1  JN661729 | Malpighiaceae | family |
| 2 | BankIt1479670 seq2  JN661730 | Astrocaryum | genus |
| 3 | BankIt1479670 seq3  JN661731 | Miconia cf. longispicata | species |
| 4 | BankIt1479670 seq4  JN661732 | Maïeta | genus |
| 5 | BankIt1479670 seq5  JN661733 | unknow | unknow |
| 6 | BankIt1479670 seq6  JN661734 | unknow | unknow |
| 7 | BankIt1479670 seq7  JN661735 | Asplundia | genus |
| 8 | BankIt1479670 seq8  JN661736 | Asplundia | genus |
| 9 | BankIt1479670 seq9  JN661737 | Asplundia | genus |
| 10 | BankIt1479670 seq10 JN661738 | Symphonia | genus |
| 11 | BankIt1479670 seq11 JN661739 | Talisia | genus |
| 12 | BankIt1479670 seq12 JN661740 | Cydista | genus |
| 13 | BankIt1479670 seq13 JN661741 | Cheiloclinium cognatum | species |
| 14 |  | Clusia | genus |
| 15 | BankIt1479670 seq15 JN661742 | Eperua falcata | species |
| 16 | BankIt1479670 seq16 JN661743 | Miconia | genus |
| 17 | BankIt1479670 seq17 JN661744 | Myristicaceae | family |
| 18 | BankIt1479670 seq18 JN661745 | Asplundia | genus |
| 19 | BankIt1479670 seq19 JN661746 | Caesalpiniaceae | subfamily |
| 20 | BankIt1479670 seq20 JN661747 | Miconia cf. longispicata | species |
| 21 | BankIt1479670 seq21 JN661748 | Vouacapoua | genus |
| 22 | BankIt1479670 seq22 JN661749 | unknow | unknow |
| 23 | BankIt1479670 seq23 JN661750 | Erisma | genus |
| 24 | BankIt1479670 seq24 JN661751 | unknow | unknow |
| 25 | BankIt1479670 seq25 JN661752 | Eperua falcata | species |
| 26 | BankIt1479670 seq26 JN661753 | Maïeta | genus |
| 27 | BankIt1479670 seq27 JN661754 | Cheiloclinium cognatum | species |
| 28 | BankIt1479670 seq28 JN661755 | Cheiloclinium | genus |
| 29 |  | unknow | unknow |
| 30 | BankIt1479670 seq30 JN661756 | Clidemia | genus |
| 31 | BankIt1479670 seq31 JN661757 | Drymonia coccinea | species |
| 32 | BankIt1479670 seq32 JN661758 | Maïeta | genus |
| 33 | BankIt1479670 seq33 JN661759 | Miconia cf. longispicata | species |
| 34 | BankIt1479670 seq34 JN661760 | Miconia | genus |
| 35 | BankIt1479670 seq35 JN661761 | Asplundia brachyphylla | species |
| 36 | BankIt1479670 seq36 JN661762 | Asplundia brachyphylla | species |
| 37 | BankIt1479670 seq37 JN661763 | Clidemia | genus |
| 38 | BankIt1479670 seq38 JN661764 | Maïeta | genus |
| 39 | BankIt1479670 seq39 JN661765 | Clusiaceae | family |
| 40 | BankIt1479670 seq40 JN661766 | Clidemia | genus |
| 41 | BankIt1479670 seq41 JN661767 | Gustavia | genus |
| 42 | BankIt1479670 seq42 JN661768 | Clidemia | genus |
| 43 | BankIt1479670 seq43 JN661769 | Odontadenia | genus |
| 44 | BankIt1479670 seq44 JN661770 | Psychotria | genus |
| 45 | BankIt1479670 seq45 JN661771 | Ebenaceae | family |
| 46 | BankIt1479670 seq46 JN661772 | Asplundia | genus |
| 47 | BankIt1479670 seq47 JN661773 | Iryanthera | genus |
| 48 | BankIt1479670 seq48 JN661774 | Naucleopsis | genus |
| 49 | BankIt1479670 seq49 JN661775 | Erisma | genus |
| 50 |  | Clusia | genus |
| 51 |  | unknow | unknow |
| 52 | BankIt1479670 seq52 JN661776 | Asplundia | genus |
| 53 | BankIt1479670 seq53 JN661777 | Symphonia | genus |
| 54 | BankIt1479670 seq54 JN661778 | Melastomataceae | family |
| 55 | BankIt1479670 seq55 JN661779 | Asplundia brachyphylla | species |
| 56 | BankIt1479670 seq56 JN661780 | unknow | unknow |
| 57 |  | unknow | unknow |
|  |  |  |  |
