# Supplementary material for: Botany, Genetics and Ethnobotany: A Crossed Investigation on the Elusive Tapir's Diet in French Guiana
Source: PLoS One. 2011 Oct 3;6(10):e25850. doi: 10.1371/journal.pone.0025850 (PMC3185057; doi:10.1371/journal.pone.0025850)
Supplement: Table S2 — List of plants browsed by tapirs. Symbols are as follows: W: species communicated by the Wayãpi indians in the ethnobotanic surveys, B: species found by the botanical study , G: species found by genetic analyses. The abundance of these plant species in the surveyed areas was categorised by the botanist experts as uncommon (UC), common (C), locally very common (LVC) and very common (VC). "DOS" and "indet." stand for "depends on the species" and "indeterminate", respectively. For plants identified only at the family level, the abundance was given for the family. The names of the taxa found by more than one method are in bold and underlined. (DOCX) [file pone.0025850.s002.docx]

**Table S2. List of plants browsed by tapirs.**

|  | |  | |  | |  |  |  |  |
| --- | --- | --- | --- | --- | --- | --- | --- | --- | --- |
| **Family** | | **Genus** | | **Species** | | | **Source** | **Type** | **Abundance** |
|  | |  | |  | |  |  |  |  |
|  | |  | |  | |  |  |  |  |
| Acanthaceae | | *Justicia* | | *cf potarensis* | | (Bremek.) Wassh. | B | herb | C |
|  | |  | | *comata* | | (L.) Lam. | B | herb | C |
| Amaryllidaceae | | *Hymenocallis* | | *tubiflora* | | Salisb. | B | herb | C |
| Annonaceae | | *Guatteria* | | *punctata* | | (Aubl.) R.A. Howard | B | tree | C |
| Apocynaceae | | *Odontadenia* | | sp. | |  | G | liana | DOS |
| Araceae | | *Anthurium* | | *rubrinervium* | | (Link) G. Don | B | herb | C |
|  | | *Philodendron* | | *grandifolium* | | (Jacq.) Schott | B | epiphytic herb | C |
|  | | *Rhodospatha* | | *oblongata* | | Poepp. | B | epiphytic herb | C |
|  | | *Spatiphyllum* | | *humboldtii* | | Schott | B | hemiepiphytic herb | C |
| Arecaceae | | *Astrocaryum* | | sp. | |  | G | palm tree | DOS |
| **Bignoniaceae** | | *Cydista* | | *sp.* | |  | G | liana | UC |
|  | | *Stizophyllum* | | *riparium* | | (Kunth) Sandwith | B | liana | ? |
|  | | indet. | | indet. | |  | B | liana | VC |
| **Clusiaceae** | | *Clusia* | | sp. | |  | G | hemiepiphytic shrub | C |
|  | | ***Symphonia*** | | *globulifera* | | L. f. | B | tree | C |
|  | |  | | sp. | |  | G | tree | C |
|  | | indet. | | indet. | |  | G | - | VC |
| **Cyclanthaceae** | | ***Asplundia*** | | ***brachyphylla*** | | Harling | W,B,G | herb | VC |
|  | |  | | *heteranthera* | | Harling | W | hemiepiphytic herb | VC |
|  | |  | | sp. | |  | G | herb | DOS |
|  | | ***Evodianthus*** | | ***funifer*** | | (Poit.) Lindm. | W,B | hemiepiphytic herb | VC |
|  | | *Ludovia* | | *lancifolia* | | Brongn. | B | epiphytic herb | VC |
| Dichapetalaceae | | *Tapura* | | *guianensis* | | Aubl. | W | tree | C |
| Dryopteridaceae | | *Didymochlaena* | | *truncata* | | (Sw.) J. Sm. | B | fern | C |
| Ebenaceae | | indet. | | indet. | |  | G | tree | C |
| **Fabaceae** | |  | |  | |  |  |  |  |
| (Caesalpiniaceae) | | ***Eperua*** | | *falcata* | | Aubl. | G | tree | VC |
|  | |  | | *rubiginosa* | | Miq. | B | tree | VC |
|  | | *Vouacapoua* | | sp. | |  | G | tree | VC |
|  | | indet. | | indet. | |  | G | - | VC |
| (Mimosaceae) | *Inga* | | *gracilifolia* | | Ducke | | B | tree | C |
| (Papilionaceae) | | *Lonchocarpus* | | *chrysophyllus* | | Kleinhoonte | W | liana | UC |
| Flacourtiaceae | | *Mayna* | | *odorata* | | Aubl. | W | shrub / tree | C |
| **Gesneriaceae** | | *Besleria* | | *insolita* | | C.V. Morton | B | herb | C |
|  | | ***Drymonia*** | | ***coccinea*** | | Aubl. | B,G | climbing herb | VC |
|  | | indet. | | indet. | |  | B,G | - | VC |
| Hippocrateaceae | | *Cheiloclinium* | | *cognatum* | | (Miers) A.C. Sm. | G | tree / liana | C |
|  | |  | |  | |  |  |  |  |
| **Lecythidaceae** | | ***Gustavia*** | | *augusta* | | L. | B | tree | LVC |
|  | |  | | sp. | |  | G | tree | LVC / C |
| Malpighiaceae | | indet. | | indet. | |  | G | liana | VC |
| **Melastomataceae** | | ***Clidemia*** | | *capitellata* | | (Bonpl.) D. Don | B | shrub | C |
|  | | ***Henriettella*** | | *caudata* | | Gleason | W | shrub | C |
|  | |  | | *flavescens* | | (Aubl.) Triana | B | tree | C |
|  | | ***Leandra*** | | ***rufescens*** | | (DC.) Cogn. | W,B | shrub | VC |
|  | |  | | *soleniflora* | | Cogn. | W | shrub | VC |
|  | | *Macrocentrum* | | *cristatum* | | (DC.) Triana | W | herb | C |
|  | | ***Maieta*** | | *guianensis* | | Aubl. | B | shrub | VC |
|  | |  | | sp. | |  | G | shrub | DOS |
|  | |  | |  | |  |  |  |  |
|  | |  | |  | |  |  |  |  |
|  | |  | |  | |  |  |  |  |
|  | |  | |  | |  |  |  |  |
|  | |  | |  | |  |  |  |  |
|  | |  | |  | |  |  |  |  |
|  | |  | |  | |  |  |  |  |
|  | |  | |  | |  |  |  |  |
|  | |  | |  | |  |  |  |  |
|  | |  | |  | |  |  |  |  |
|  | |  | |  | |  |  |  |  |
|  | |  | |  | |  |  |  |  |

**Table 2** (suite)

|  |  |  |  |  |  |  |
| --- | --- | --- | --- | --- | --- | --- |
| **Family** | **Genus** | **Species** | | **Source** | **Type** | **Abundance** |
|  |  |  |  |  |  |  |
|  |  |  |  |  |  |  |
|  | ***Miconia*** | *ampla* | Triana | W | tree | UC |
|  |  | *bracteata* | (AP De Candolle) Triana | B | shrub | C |
|  |  | *ceramicarpa* | (DC.) Triana | W | shrub | VC |
|  |  | ***cf longispicata*** | Triana | B,G | shrub | UC |
|  |  | *diaphanea* | Gleason | B | shrub | C |
|  |  | *lateriflora* | Cogn. | W | shrub | C |
|  |  | *prasina* | (Sw.) DC. | W | tree | C |
|  |  | *sastrei* | Wurdack | B | shrub | UC |
|  |  | *serrulata* | (DC.) Naudin | W | shrub | C |
|  |  | sp. |  | G | - | DOS |
|  |  | *trinervia* | (OP Swartz) D Don ex Loudon | W | shrub | UC |
|  | indet. | indet. |  | G | - | VC |
| **Moraceae** | *Ficus* | *insipida* | Wildl. | B | tree | C |
|  | *Naucleopsis* | sp. |  | G | tree | DOS |
|  | *Perebea* | *guianensis* | Aubl. | B | tree | C |
| Myristicaceae | *Iryanthera* | sp. |  | G | tree | DOS |
|  | indet. | indet. |  | G | - | VC |
| Polygalaceae | *Moutabea* | *guianensis* | Aubl. | B | shrub / liana | VC |
| Polygonaceae | *Coccoloba* | *lucidula* | Benth. | B | shrub | UC |
| Rapateaceae | *Rapatea* | *paludosa* | Aubl. | B | forb | LVC |
| **Rubiaceae** | *Carapichea* | *guianense* | Aubl. | W | shrub | UC |
|  | ***Faramea*** | *guianensis* | (Aubl.) Bremek. | B | shrub | VC |
|  |  | *multiflora* | A. Rich. ex DC. | W | tree | C |
|  | ***Psychotria*** | *capitata* | Ruiz & Pav. | W | shrub | VC |
|  |  | *cupularis* | (Müll. Arg.) Standl. | W | shrub | VC |
|  |  | *mapourioides* | DC. | W | shrub / tree | C |
|  |  | *medusula* | Müll. Arg. | W | shrub | C |
|  |  | ***microbotrys*** | Ruiz ex Standl. | W,B | shrub | C |
|  |  | *racemosa* | (Aubl.) Raeusch. | W | shrub | VC |
|  |  | spp. |  | B,G | - | DOS |
|  | *Rudgea* | *guianensis* | (A. Rich.) Sandwith | W | shrub | UC |
|  | indet. | indet. |  | B | - | VC |
| Rutaceae | indet. | indet. |  | B | - | C |
| **Sapindaceae** | *Allophylus* | *leucoclados* | Radlk. | W | tree | UC |
|  | *Paullinia* | *rugosa* | Benth. ex Radlk. | B | liana | UC |
|  | *Serjania* | *oblongifolia* | Radlk. | B | liana | UC |
|  | *Talisia* | sp. |  | G | tree | DOS |
| Smilacaceae | *Smilax* | sp. |  | B | liana | DOS |
| Sterculiaceae | *Sterculia* | *pruriens* | (Aubl.) K. Schum. | B | tree | VC |
| Symplocaceae | *Symplocos* | *martinicensis* | Jacq. | B | tree | UC |
| Tectariaceae | *Tectaria* | *trifolia* | (Alderw.) C. Chr. | B | forb (fern) | VC |
| Thelypteridaceae | *Thelypteris* | *glandulosa* | (Desv.) Proctor | B | forb (fern) | C |
|  |  | *leprieuri* | (Hook.) R.M. Tryon | B | forb (fern) | C |
|  |  | *macrophylla* | (Kunze) C.V. Morton | B | forb (fern) | C |
| Thurniaceae | *Thurnia* | *sphaerocephala* | (Rudge) Hook. f. | B | aquatic sedge | LVC |
| Ulmaceae | *Celtis* | *iguanaea* | (Jacq.) Sarg. | B | shrub | UC |
| Violaceae | *Rinorea* | *riana* | Kuntze | B | tree | VC |
| Vochysiaceae | *Erisma* | sp. | Rudge | G | tree | DOS |
| Zingiberaceae | *Renealmia* | *monosperma* | Miq. | B | forb | C |
| Indeterminate | Indet. | Indet. |  | B,G | - | - |
|  |  |  |  |  |  |  |
|  |  |  |  |  |  |  |
|  |  |  |  |  |  |  |
|  |  |  |  |  |  |  |
|  |  |  |  |  |  |  |
|  |  |  |  |  |  |  |
|  |  |  |  |  |  |  |
|  |  |  |  |  |  |  |
|  |  |  |  |  |  |  |

Symbols are as follows: W: species communicated by the Wayãpi indians in the ethnobotanic surveys, B: species found by the botanical study , G: species found by genetic analyses. The abundance of these plant species in the surveyed areas was categorised by the botanist experts as uncommon (UC), common (C), locally very common (LVC) and very common (VC). "DOS" and "indet." stand for "depends on the species" and "indeterminate", respectively. For plants identified only at the family level, the abundance was given for the family. The names of the taxa found by more than one method are in bold and underlined.
