# Supplementary material for: Botany, Genetics and Ethnobotany: A Crossed Investigation on the Elusive Tapir's Diet in French Guiana
Source: PLoS One. 2011 Oct 3;6(10):e25850. doi: 10.1371/journal.pone.0025850 (PMC3185057; doi:10.1371/journal.pone.0025850)
Supplement: Table S3 — List of fruits eaten by tapirs. Symbols are as follows: W: species communicated by the Wayãpi indians and D: species identified from the macroscopic analysis of fruit remains in dung. For the latter, we also indicated whether the seeds were intact (int.) or damaged (dam.) and the month of collection. The fructification periods observed during the ethnobotanical survey are indicated with *.The names of the taxa found by several approaches are in bold and underlined. Indeterminate species are indicated by "indet.". (DOCX) [file pone.0025850.s003.docx]

**Table S3: List of fruits eaten by tapirs.**

|  |  |  |  |  |  |  |
| --- | --- | --- | --- | --- | --- | --- |
| **Family** | **Genus** | **Species** | | **Source** | **Seed condition** | **Period** |
|  |  |  |  |  |  |  |
| **Anacardiaceae** | ***Spondias*** | ***mombin*** | L. | W,D | int. | Dec-Apr |
|  | *Tapirira* | *guianensis* | Aubl. | D | int. | Apr |
|  | *Thyrsodium* | *puberulum* | J.D. Mitch. & D.C. Daly | W |  |  |
| Annonaceae | *cf Duguetia* | sp. |  | D | dam. | Jul |
|  | *Unonopsis* | sp. |  | D | dam. | Mar |
| **Apocynaceae** | *Geissospermum* | *laeve* | (Vell.) Miers | D | int. or dam. | Feb-Jul |
|  | *Lacmellea* | *aculeata* | (Ducke) Monach. | W |  | Apr-May* |
|  | *Pacouria* | *guianensis* | Aubl. | D | int. or dam. | Jan, Mar-Apr |
|  | *Parahancornia* | *fasciculata* | (Poir.) Benoist | W |  | Feb-Mar* |
| Arecaceae | *Astrocaryum* | *paramaca* | Mart. | D | dam. | Feb-Apr, Jul |
|  | *Attalea* | *attaleoides* | (Barb. Rodr.) Wess. Boer | D | dam. | Jul |
|  | *cf Bactris* | sp. |  | D | int. (immature) | Jul |
|  | *Euterpe* | *oleracea* | Mart. | D | int. or dam. | Apr-Jul |
|  | *Oenocarpus* | *bataua* | Mart. | D | dam. | Jun |
| Burseraceae | *Protium* | *gallicum* | D.C. Daly | W |  |  |
| **Caricaceae** | ***Jacaratia*** | ***spinosa*** | (Aubl.) A. DC. | D, W | int. or dam. | Jun-Jul |
| Clusiaceae | *Rheedia* | *madruno* | (Kunth) Planch. & Triana | W |  |  |
| Clusiaceae or Lecythidaceae | indet. | indet. |  | D | int. | Apr |
| Combretaceae | *Buchenavia* | *capitata* | (Vahl) Eichler | D | int. | Aug |
|  |  | *guianensis* | Alwan & Stace | D | int. | Sep |
| Cyperaceae | indet*.* | indet*.* |  | D | int. | Apr, Jul |
| **Fabaceae** |  |  |  |  |  |  |
| (Caesalpiniaceae) | *Swartzia* | *polyphylla* | DC. | D | dam. | Apr |
| (Mimosaceae) | *Stryphnodendron* | *cf polystachyum* | (Miq.) Kleinhoonte | D | int. | Jul |
|  |  | sp. |  | D | dam. | Jul |
| (Papilionaceae) | *Dipteryx* | *cf punctata* | (S.F. Blake) Amshoff | D | int. | Jun-Jul |
| Hippocrateaceae | *Salacia* | sp. |  | D | dam. | Apr |
| **Humiriaceae** | *Humiria* | *balsamifera* | J. St.-Hil. | D | int. | Jul |
|  | ***Sacoglottis*** | *cydonioïdes* | Cuatrec. | D | int. | Feb-Apr |
|  |  | *guianensis* | Benth. | W |  |  |
| Lecythidaceae | *Lecythis* | sp. |  | D | dam. | Apr |
| Malpighiaceae | *Byrsonima* | sp. |  | D | int. | Jul |
| Melastomataceae | *Mouriri* | *collocarpa* | Ducke | D | int. | Mar, Jun |
|  |  | *crassifolia* | Ducke | D | int. or dam. | Apr-Jul |
| Meliaceae | *Carapa* | sp. |  | D | dam. | Mar |
|  |  |  |  |  |  |  |
|  |  |  |  |  |  |  |
|  |  |  |  |  |  |  |

**Table S3** (suite)

|  |  |  |  |  |  |  |
| --- | --- | --- | --- | --- | --- | --- |
| **Family** | **Genus** | **Species** | | **Source** | **Seed condition** | **Period** |
|  |  |  |  |  |  |  |
| **Moraceae** | ***Bagassa*** | ***guianensis*** | Aubl. | W,D | int. | Mar |
|  | *Ficus* | spp. |  | D | int. | Aug-Nov |
|  | ***Helicostylis*** | *tomentosa* | (Poepp. & Endl.) Rusby | W |  | Apr-Jun* |
|  |  | sp. |  | D | int. or dam. | Apr-Jul |
|  | *Perebea* | *mollis* | (Poepp. & Endl.) Huber | W |  | Mar-Apr* |
| Olacaceae | *Heisteria* | *scandens* | Duke | D | int. or dam. | Apr |
| Poaceae | indet*.* | indet*.* |  | D | int. | Sep |
| Polygalaceae | *Moutabea* | sp. |  | D | dam. | Apr |
| Quiinaceae | *Lacunaria* | *crenata* | (Tul.) A.C. Sm. | D | int. | Jul |
| **Rubiaceae** | ***Genipa*** | *americana* | L. | W |  | Jun-Jul* |
|  |  | sp. |  | D | int. | Mar, Jun-Jul |
|  | *Psychotria* | *kappleri* | (Miq.) Müll. Arg. ex Benoist | D | dam. | Apr |
|  |  | sp. *1* |  | D | dam. | Jul |
|  |  | sp. *2* |  | D | int. | Jun |
|  |  | sp. *3* |  | D | int. | Jul |
|  | indet*.* | indet |  | D | int. | Jul |
| **Sapotaceae** | *Chrysophyllum* | *pomiferum* | (Eyma) T.D. Penn. | D | int. or dam. | Jun |
|  | *Diploon* | *cuspidatum* | (Hoehne) Cronquist | D | int. | Apr |
|  | *Ecclinusa* | *guianensis* | Eyma | W |  | Apr-May* |
|  | *Manilkara* | *bidentata* | (A. DC.) A. Chev. | W |  | Dec-Jan* |
|  | ***Micropholis*** | ***melinoniana*** | Pierre | W,D | int. | Mar |
|  |  | sp. |  | D | int. or dam. | Apr |
|  | ***Pouteria*** | *egregia* | Sandwith | D | dam. | Apr |
|  |  | *franciscana* | Baehni | W |  | Jan-Apr* |
|  |  | sp. *1* |  | D | int. or dam. | Apr |
|  |  | sp. *2* |  | D | dam. | Apr |
|  | ***Pradosia*** | ***ptychandra*** | (Eyma) T.D. Penn. | W,D | dam. | Apr |
|  | indet*.* | indet*.* |  | D | int. or dam. | Mar-Jul |
| Siparunaceae | *Siparuna* | *cristata* | (Poepp. & Endl.) A. DC. |  |  |  |
|  |  | *OR decipiens* | (Tul.) A. DC. | W |  | Mars-Apr |
| Sterculiaceae | *Theobroma* | *cacao* | L. | W |  |  |
| Urticaceae | *Cecropia* | *obtusa* | Aubl. | D | int. | Aug, Nov |
|  |  | *sciadophylla* | Mart. | D | int. | Nov |
|  | *Pourouma* | sp. |  | D | dam. | Mar |
| Indet. | indet*.* | indet*.* |  | D | int. or dam. | Feb-Aug |
|  |  |  |  |  |  |  |

Symbols are as follows: W: species communicated by the Wayãpi indians and D: species identified from the macroscopic analysis of fruit remains in dung. For the latter, we also indicated whether the seeds were intact (int.) or damaged (dam.) and the month of collection. The fructification periods observed during the ethnobotanical survey are indicated with *.The names of the taxa found by several approaches are in bold and underlined. Indeterminate species are indicated by "indet.".
