# Supplementary material for: Botany, Genetics and Ethnobotany: A Crossed Investigation on the Elusive Tapir's Diet in French Guiana
Source: PLoS One. 2011 Oct 3;6(10):e25850. doi: 10.1371/journal.pone.0025850 (PMC3185057; doi:10.1371/journal.pone.0025850)
Supplement: Table S4 — List of plants eaten by lowland tapirs in South America. (DOCX) [file pone.0025850.s004.docx]

**Table S4. List of plants eaten by lowland tapirs in South America**

| **Family** | **Species** | **Eaten part** | **References** |
| --- | --- | --- | --- |
|  |  |  |  |
|  |  |  |  |
|  |  |  |  |
| Acanthaceae | *Justicia cf. potarensis (Bremek.) Wassh.* | b | TS |
|  | *Justicia comata (L.) Lam.* | b | TS |
|  | *Odontonema bracteolatum (Jacq.) Kuntze* | b | [23] |
| Amaryllidaceae | *Hymenocallis tubiflora Salisb.* | b | TS |
| Anacardiaceae | *Anacardium giganteum W. Hancock ex Engl.* | f | [23,39] |
|  | *Mangifera indica L.* | f | [39,87] |
|  | *Spondias mombin L.* | f | TS,[23,39,41,43,83] |
|  |  | b | [43] |
|  | *Spondias* sp. | f | [25] |
|  | *Tapirira guianensis Aubl.* | f | TS |
|  | *Thyrsodium puberulum J.D. Mitch. & D.C. Daly* | f | TS |
| Annonaceae | *Anaxagorea* sp. | b | [23] |
|  | *Annona* spp. | f | [43,83,89] |
|  | *Duguetia pycnastera Sandwith* | b | [23] |
|  | *Duguetia riparia Huber* | f | [83] |
|  | *cf Duguetia* sp. | f | TS |
|  | *Fusaea longifolia (Aubl.) Saff.* | f | [41,83] |
|  | *Guatteria punctata (Aubl.) R.A. Howard* | b | TS |
|  | *Guatteria* sp. | b | [23] |
|  | *Onychopetalum periquino (Rusby) D.M. Johnson & N.A. Murray* | f | [83] |
|  | *Oxandra euneura Diels* | b | [43] |
|  | *Oxandra xylopioides Diels* | f | [83] |
|  | *Porcelia nitidifolia Ruiz & Pav.* | f | [83] |
|  | *Rollinia* spp. | f | [83,89] |
|  | *Unonopsis* sp. | f | TS |
|  | *Xylopia calophylla R.E. Fr.* | b | [23] |
|  | indet. | f | [25] |
| Apocynaceae | *Ambelania acida Aubl.* | f | [41] |
|  | *Aspidosperma cf. marcgravianum Woodson* | b | [23] |
|  | *Couma macrocarpa Barb. Rodr.* | f | [23,43] |
|  | *Geissospermum laeve (Vell.) Miers* | f | TS,[41] |
|  | *Lacmellea aculeata (Ducke) Monach.* | f | TS |
|  | *Macoubea guianensis Aubl.* | f | [83] |
|  | *Odontadenia* sp. | b | TS |
|  | *Pacouria guianensis Aubl.* | f | TS,[41] |
|  | *Parahancornia fasciculata (Poir.) Benoist* | f | TS,[41] |
|  | *Rauvolfia paraensis Ducke* | f | [41] |
|  | *Tabernaemontana flavicans Willd. ex Roem. & Schult.* | b | [43] |
|  | *Tabernaemontana sananho Ruiz & Pav.* | b | [23] |
|  |  | f | [23] |
|  | *Tabernaemontana siphilitica Leeuwenb.* | b | [43] |
|  | *Tabernaemontana* sp. | b | [23] |
| Aquifoliaceae | *Ilex dumosa Reissek* | b | [67] |
|  | *Ilex pseudobuxus Reissek* | b | [67] |
| Araceae | *Anthurium brevipedunculatum Madison* | b | [43] |
|  | *Anthurium clavigerum Poepp.* | b | [23] |
|  | *Anthurium rubrinervium (Link) G. Don* | b | TS |
|  | *Heteropsis flexuosa (Kunth) G.S. Bunting* | b | [23] |
|  | *Heteropsis* sp*.* | b | [43] |
|  | *Heteropsis spruceana Schott* | f | [83] |
|  | *Philodendron bipinnatifidum Schott ex Endl.* | b | [67] |
|  | *Philodendron elaphoglossoides Schott* | b | [43] |
|  | *Philodendron fragrantissimum (Hook.) Kunth* | b | [23] |
|  | *Philodendron grandifolium (Jacq.) Schott* | b | TS |
|  | *Philodendron hylaeae G.S. Bunting* | b | [23] |
|  | *Philodendron rudgeanum Schott* | b | [43] |
|  | *Philodendron* sp. | b | [23] |
|  | *Rhodospatha oblongata Poepp.* | b | TS,[23] |
|  | *Spatiphyllum humboldtii Schott* | b | TS |
|  | indet. | f | [25,42] |
| Araliaceae | *Dendropanax arboreus (L.) Decne. & Planch.* | b | [43] |
| Arecaceae | *Astrocaryum murumuru Mart.* | f | [83] |
|  | *Astrocaryum paramaca Mart.* | f | TS |
|  | *Astrocaryum* sp. | b | TS |
|  |  | f | [39,41] |
|  | *Attalea attaleoides (Barb. Rodr.) Wess. Boer* | f | TS |
|  | *Attalea phalerata Mart. ex Spreng.* | f | [85] |
|  | *Attalea* sp. | f | [43] |
|  | *Bactris cf. hirta Mart.* | f | [83] |
|  | *cf. Bactris* sp. | f | TS |
|  | *Butia capitata (Mart.) Becc.* | f | [67] |
|  | *Euterpe edulis Mart.* | f | [87] |
|  | *Euterpe oleracea Mart.* | f | TS |
|  | *Jessenia bataua (Mart.) Burret* | f | [23,41] |
|  | *Jessenia* sp. | f | [42] |
|  | *Mauritia flexuosa L. f.* | f | [25,39,41-43,83] |
|  | *Maximiliana maripa (Aubl.) Drude* | b | [23] |
|  |  | f | [38,39] |
|  | *Oenocarpus bacaba Mart.* | f | [39] |
|  | *Oenocarpus bataua Mart.* | f | TS,[23,43,83] |
|  | *Oenocarpus mapora H. Karst.* | f | [83] |
|  | *Sheelea* sp. | f | [25,42] |
|  | *Syagrus oleracea (Mart.) Becc.* | f | [87] |
|  | *Syagrus romanzoffiana (Cham.) Glassman* | f | [84,86-89] |
| Asteraceae | *Eupatorium* spp. | b | [67] |
|  | *Vernonia scorpioides (Lam.) Pers.* | b | [67] |
|  | *Vernonia* sp. | b | [67] |
|  | *Wedelia ambigens S.F. Blake* | b | [23] |
| Bignoniaceae | *Arrabidaea bilabiata (Sprague) Sandwith* | b | [43] |
|  | *Cydista* sp. | b | TS |
|  | *Stizophyllum riparium (Kunth) Sandwith* | b | TS |
|  | indet. | b | TS |
| Blechnaceae | *Blechnum serrulatum Rich.* | b | [67] |
|  | *Salpichlaena hookeriana (Kuntze) Alston* | b | [43] |
| Bombacaceae | *Catostemma commune Sandwith* | b | [23] |
|  | *Ceiba pentandra (L.) Gaertn.* | f | [83] |
|  | *Matisia bracteolosa Ducke* | b | [43] |
|  | *Matisia cordata Bonpl.* | f | [83] |
|  | *Ochroma pyramidale (Cav. ex Lam.) Urb.* | f | [83] |
|  | *Pachira aquatica Aubl.* | f | [83] |
| Boraginaceae | *Cordia curassavica (Jacq.) Roem. & Schult.* | b | [67] |
|  | *Cordia monosperma (Jacq.) Roem. & Schult.* | b | [67] |
|  | *Cordia* sp. | f | [41] |
| Bromeliaceae | indet. | f | [39] |
|  | *Tillandsia usneoides (L.) L.* | b | [67] |
| Burseraceae | *Dacryodes peruviana (Loes.) H.J. Lam* | b | [23] |
|  |  | f | [23] |
|  | *Protium cf. amazonicum (Cuatrec.) D.C. Daly* | f | [83] |
|  | *Protium crassipetalum Cuatrec.* | b | [23] |
|  | *Protium crenatum Sandwith* | b | [23] |
|  |  | f | [23] |
|  | *Protium gallicum D.C. Daly* | f | TS |
|  | *Protium nitidifolium (Cuatrec.) D.C. Daly* | b | [23] |
|  |  | f | [23] |
|  | *Protium* spp. | f | [83] |
|  | *Tetragastris panamensis (Engl.) Kuntze* | f | [39] |
| Capparidaceae | *Capparis macrophylla Kunth* | b | [43] |
| Caricaceae | *Jacaratia digitata (Poepp. & Endl.) Solms* | f | [83] |
|  | *Jacaratia spinosa (Aubl.) A. DC.* | f | TS,[41] |
|  | *Jacaratia* sp. | f | [43] |
| Celastraceae | *Maytenus* sp. | f | [43] |
| Chrysobalanaceae | *Hirtella racemosa Lam.* | f | [83] |
|  | *Hirtella* sp. | f | [83] |
|  | *Licania octandra (Hoffmanns. ex Roem. & Schult.) Kuntze* | b | [23] |
|  | *Licania pallida Spruce ex Sagot* | b | [23] |
|  | *Licania* sp. | f | [43] |
|  | *Parinari excelsa Sabine* | f | [23,41] |
|  | *indet.* | f | [25,42] |
| Clusiaceae | *Chrysochlamys* sp. | b | [43] |
|  | *Clusia parvifolia Maguire* | b | [67] |
|  | *Clusia* spp. | f | TS,[83] |
|  | *Garcinia acuminata Pierre* | b | [43] |
|  | *Garcinia macrophylla Mart.* | b | [23] |
|  |  | f | [23] |
|  | *Moronobea coccinea Aubl.* | b | [43] |
|  | *Rheedia madruno (Kunth) Planch. & Triana* | f | TS |
|  | *Symphonia globulifera L. f.* | b | TS |
|  |  | f | [41] |
|  | *Symphonia* sp. | b | TS |
|  | *Vismia japurensis Reichardt* | b | [23] |
|  | *Vismia* sp. | f | [83] |
|  | indet. | b | TS |
| Combretaceae | *Buchenavia capitata (Vahl) Eichler* | f | TS |
|  | *Buchenavia guianensis Alwan & Stace* | f | TS |
|  | *Buchenavia* sp. | f | [25] |
|  | *Combretum fruticosum (Loefl.) Stuntz* | b | [43] |
|  | *Combretum laurifolium Engl.* | b | [43] |
|  | *Combretum laxum Jacq.* | b | [43] |
| Convolvulaceae | *Maripa* sp. | b | [43] |
| Cucurbitaceae | *Cayaponia* sp. | f | [41] |
| Cyclanthaceae | *Asplundia brachyphylla Harling* | b | TS |
|  | *Asplundia heteranthera Harling* | b | TS |
|  | *Asplundia* sp. | b | TS |
|  | *Evodianthus funifer (Poit.) Lindm.* | b | TS,[43] |
|  | *Ludovia lancifolia Brongn.* | b | TS |
|  | indet. | b | [23] |
| Cyperaceae | *Scleria macrophylla J. Presl & C. Presl* | f | [83] |
|  | *Senefeldera inclinata Müll. Arg.* | f | [83] |
|  | indet. | f | TS,[83] |
| Dichapetalaceae | *Tapura guianensis Aubl.* | b | TS |
| Dillienaceae | *Doliocarpus dentatus (Aubl.) Standl.* | b | [23] |
| Dryopteridaceae | *Didymochlaena truncata (Sw.) J. Sm.* | b | TS |
| Ebenaceae | *Diospyros poeppigiana A. DC.* | b | [43] |
|  | *Diospyros subrotata Hiern* | b | [43] |
|  | indet. | b | TS |
| Elaeocarpaceae | *Sloanea grandiflora Sm.* | b | [23] |
| Erythroxylaceae | *Erythroxylum amplifolium (Mart.) O.E. Schulz* | b | [67] |
| Euphorbiaceae | *Acalipha* sp. | b | [43] |
|  | *Alchornea triplinervia (Spreng.) Müll. Arg.* | b | [23,67] |
|  | *Aparisthmium cordatum Baill.* | b | [23] |
|  | *Conceveiba rhytidocarpa Müll. Arg.* | b | [43] |
|  | *Didymocistus chrysadenius Kuhlm.* | b | [43] |
|  | *Mabea cf. piriri Aubl.* | b | [23] |
|  | *Mabea elata Steyerm.* | b | [43] |
| Fabaceae Caesalpiniaceae | *Bauhinia guianensis Aubl.* | b | [23] |
|  | *Cassia moschata Kunth* | f | [39] |
|  | *Copaifera langsdorffii Desf.* | f | [87] |
|  | *Eperua falcata Aubl.* | b | TS |
|  | *Eperua rubiginosa Miq.* | b | TS |
|  | *Hymenaea oblongifolia Huber* | f | [83] |
|  | *Hymenaea* sp. | f | [43] |
|  | *Macrolobium angustifolium (Benth.) R.S. Cowan* | b | [43] |
|  | *Senna* sp. | f | [83,89] |
|  | *Swartzia polyphylla DC.* | f | TS |
|  | *Swartzia* sp. | f | [39] |
|  | *Vouacapoua* sp. | b | TS |
|  | *indet.* | b | TS |
| Fabaceae Mimosaceae | *Acacia* sp. | f | [83] |
|  | *Enterolobium contortisiliquum (Vell.) Morong* | f | [87] |
|  | *Enterolobium schomburgkii (Benth.) Benth.* | f | [39] |
|  | *Inga alba (Sw.) Willd.* | b | [43] |
|  | *Inga dumosa Benth.* | b | [43] |
|  | *Inga capitata Desv.* | b | [23,43] |
|  | *Inga semialata (Vell.) Mart.* | b | [43] |
|  | *Inga gracilifolia Ducke* | b | TS |
|  | *Inga oerstediana Benth. ex Seem.* | b | [23] |
|  |  | f | [23] |
|  | *Inga* sp. | b | [23] |
|  |  | f | [25,87] |
|  | *Mimosa sp.* | f | [83] |
|  | *Parkia pendula (Willd.) Benth. ex Walp.* | f | [83] |
|  | *Stryphnodendron cf. polystachyum (Miq.) Kleinhoonte* | f | TS,[41] |
|  | *Stryphnodendron* sp. | f | TS |
|  | *Zygia inaequalis (Humb. & Bonpl. ex Willd.) Pittier* | b | [23] |
| Fabaceae Papilionaceae | *Derris* sp. | b | [23] |
|  | *Desmodium* sp. | f | [39] |
|  | *Diplotropis purpurea (Rich.) Amshoff* | b | [23] |
|  | *Dipteryx odorata (Aubl.) Willd.* | f | [23] |
|  | *Dipteryx cf punctata (S.F. Blake) Amshoff* | b | [23] |
|  |  | f | TS,[23] |
|  | *Lonchocarpus chrysophyllus Kleinhoonte* | b | TS |
|  | *Machaerium floribundum Benth.* | b | [43] |
|  | *Machaerium* sp. | b | [23] |
|  | *Ormosia* sp. | f | [83] |
|  | indet. | f | [42] |
| Flacourtiaceae | *Hasseltia floribunda Kunth* | b | [43] |
|  | *Laetia procera (Poepp.) Eichler* | b | [23] |
|  | *Mayna odorata Aubl.* | b | TS |
|  | *Ryania speciosa Vahl* | b | [43] |
|  | *Tetrathylacium macrophyllum Poepp.* | b | [43] |
|  | indet. | f | [83] |
| Gesneriaceae | *Besleria insolita C.V. Morton* | b | TS |
|  | *Drymonia coccinea Aubl.* | b | TS |
|  | *indet.* | b | TS |
| Hippocrataceae | *Salacia* sp. | b | [43] |
|  | *cf. Cheiloclignum* sp. | f | [41] |
|  | *Cheiloclinium cognatum (Miers) A.C. Sm.* | b | TS |
|  | *Cuervea kappleri (Miq.) A.C. Sm.* | b | [23] |
|  | *Salacia* sp. | f | TS |
| Hugoniaceae | *Hebepetalum humiriifolium (Planch.) Benth.* | f | [83] |
| Humiriaceae | *Humiria balsamifera J. St.-Hil.* | f | TS |
|  | *Sacoglottis cydonioïdes Cuatrec.* | f | TS,[41] |
|  | *Sacoglottis guianensis Benth.* | f | TS |
| Icacinaceae | *Calatola* sp. | f | [25,83] |
|  | *Discophora guianensis Miers* | b | [23] |
|  | *Humirianthera ampla (Miers) Baehni* | b | [43] |
|  | *in*det. spp. | f | [23] |
| Lauraceae | *Ocotea pulchella (Nees) Mez* | b | [67] |
|  | indet. | b | [23] |
| Lecythidaceae | *Couroupita guianensis Aubl.* | f | [83] |
|  | *Eschweilera tessmannii R. Knuth* | b | [43] |
|  | *Gustavia augusta L.* | b | TS |
|  | *Gustavia coriacea S.A. Mori* | b | [23] |
|  |  | f | [23] |
|  | *Gustavia* sp. | b | TS |
|  | *Lecythis chartacea O. Berg* | b | [23] |
|  | *Lecythis* sp. | f | TS |
| Loganiaceae | *Strychnos* sp. | f | [41] |
| Malpighiaceae | *Byrsonima laevigata (Poir.) DC.* | f | [41] |
|  | *Byrsonima* sp. | b | [23] |
|  |  | f | TS,[83] |
|  | *indet.* | b | TS |
| Malvaceae | *Sida* sp. | b | [67] |
| Marantaceae | indet. | b | [23] |
| Melastomataceae | *Bellucia aff. pentamera Naudin* | b | [23] |
|  | *Bellucia grossulariodes (L.) Triana* | f | [41] |
|  | *Clidemia capitellata (Bonpl.) D. Don* | b | TS |
|  | *Henriettea lasiostylis Pilg.* | b | [43] |
|  | *Henriettella caudata Gleason* | b | TS |
|  | *Henriettella flavescens (Aubl.) Triana* | b | TS |
|  | indet. spp. | b | TS,[23] |
|  | indet. spp. | f | [89] |
|  | *Leandra rufescens (DC.) Cogn.* | b | TS |
|  | *Leandra soleniflora Cogn.* | b | TS |
|  | *Loreya mespiloides Miq.* | b | [43] |
|  | *Macrocentrum cristatum (DC.) Triana* | b | TS |
|  | *Maieta guianensis Aubl.* | b | TS,[23] |
|  | *Maieta* sp. | b | TS |
|  | *Miconia amazonica Triana* | b | [43] |
|  | *Miconia ampla Triana* | b | TS |
|  | *Miconia bracteata (AP De Candolle) Triana* | b | TS |
|  | *Miconia ceramicarpa (DC.) Triana* | b | TS |
|  | *Miconia cf longispicata Triana* | b | TS |
|  | *Miconia diaphanea Gleason* | b | TS |
|  | *Miconia eriocalyx Cogn.* | b | [43] |
|  | *Miconia laterifolia Cogn.* | b | TS |
|  | *Miconia ligustroides (DC.) Naudin* | b | [67] |
|  | *Miconia mazanana J.F. Macbr.* | b | [43] |
|  | *Miconia nervosa (Sm.) Triana* | b | [43] |
|  | *Miconia prasina (Sw.) DC.* | b | TS |
|  | *Miconia sastrei Wurdack* | b | TS |
|  | *Miconia serrulata (DC.) Naudin* | b | TS |
|  | *Miconia splendens (Sw.) Griseb.* | b | [43] |
|  | *Miconia* spp. | b | TS,[23] |
|  |  | f | [83] |
|  | *Miconia ternatifolia Triana* | b | [43] |
|  | *Miconia trinervia (OP Swartz) D Don ex Loudon* | b | TS |
|  | *Mouriri collocarpa Ducke* | f | TS,[41] |
|  | *Mouriri crassifolia Ducke* | f | TS |
|  | *Mouriri sagotiana Triana* | b | [23] |
|  | *Tibouchina urvilleana (DC.) Cogn.* | b | [67] |
|  | *Tococa capitata Trail ex Cogn.* | b | [43] |
|  | *Tococa caudata Markgr.* | b | [43] |
|  | *Tococa coronata Benth.* | b | [43] |
|  | *Tococa guianensis Aubl.* | b | [43] |
|  | *Tococa setifera Pilg.* | b | [43] |
| Meliaceae | *Carapa* sp. | f | TS |
|  | *Trichilia pallida Sw.* | b | [43] |
| Menispermaceae | *Anomospermum cf. reticulatum (Mart.) Eichler* | f | [23,83] |
|  | *Elephantomene eburnea Barneby & Krukoff* | f | [41] |
|  | indet. | b | [23] |
|  | indet. | f | [23,25,42] |
| Moraceae | *Bagassa guianensis Aubl.* | f | TS,[39,41] |
|  | *Brosimum alicastrum Sw.* | f | [83] |
|  | *Brosimum parinarioides Ducke* | f | [41] |
|  | *Clarisia racemosa Ruiz & Pav.* | f | [83] |
|  | *Coussapoa sp.* | f | [25] |
|  | *Ficus insipida Wildl.* | b | TS |
|  |  | f | [43] |
|  | *Ficus spp.* | f | TS,[23,39,41,83,87] |
|  | *Helicostylis* sp. | f | TS |
|  | *Helicostylis tomentosa (Poepp. & Endl.) Rusby* | f | TS,[23,41,83] |
|  | *Naucleopsis* sp. | b | TS,[43] |
|  | *Perebea guianensis Aubl.* | b | TS |
|  | *Perebea mollis (Poepp. & Endl.) Huber* | f | TS |
|  | *Perebea* spp. | f | [83] |
|  | *Pseudolmedia laevis (Ruiz & Pav.) J.F. Macbr.* | b | [23] |
|  |  | f | [23] |
|  | *Sorocea hirtella Mildbr.* | b | [43] |
|  | *Sorocea muriculata Miq.* | b | [23] |
| Musaceae | *Heliconia acuminata Rich.* | b | [23] |
| Myristicaceae | indet. | b | TS |
|  | *Iryanthera* sp*.* | b | TS |
|  | *Iryanthera ulei Warb.* | b | [23] |
|  | *Otoba glycycarpa (Ducke) W.A. Rodrigues & T.S. Jaramillo* | b | [43] |
|  | *Otoba parvifolia (Markgr.) A.H. Gentry* | b | [43] |
|  | *Virola elongata (Benth.) Warb.* | b | [43] |
| Myrtaceae | *Calyptranthes multiflora Poepp. ex O. Berg* | b | [23] |
|  | *Eugenia feijoi O. Berg* | b | [23] |
|  | *Eugenia cf. florida DC.* | f | [83] |
|  | *Eugenia myrobalana DC.* | b | [43] |
|  | *Myrcia paivae O. Berg* | b | [43] |
|  | *Myrcia rostrata DC.* | b | [67] |
|  | *Myrciaria* sp. | b | [43] |
|  | *Psidium cattleianum Sabine* | f | [67] |
|  | *Psidium guayava Raddi* | b | [67] |
|  |  | f | [87] |
|  | *Psidium myrtoides O. Berg* | f | [89] |
|  | *Psidium* sp. | f | [39,83] |
| Nyctaginaceae | *Neea floribunda Poepp. & Endl.* | b | [43] |
|  | *Neea spruceana Heimerl* | b | [43] |
| Ochnaceae | *Ouratea aromatica J.F. Macbr.* | b | [43] |
|  | *Ouratea* sp. | b | [43,67] |
| Olacaceae | *Heisteria cauliflora cauliflora Sm.* | b | [43] |
|  | *Heisteria scandens Duke* | f | TS |
| Onagraceae | *Ludwigia multinervia (Hook. & Arn.) Ramamoorthy* | b | [67] |
| Passifloraceae | *Passiflora* sp. | f | [39] |
| Piperaceae | *Piper* sp. | b | [67] |
|  |  | f | [41] |
| Poaceae | *indet.* | f | TS,[25,39,89] |
|  | *Olyra* sp. | f | [83] |
| Polygalaceae | *Moutabea aculeata (Ruiz & Pav.) Poepp. & Endl.* | f | [83] |
|  | *Moutabea guianensis Aubl.* | b | TS |
|  | *Moutabea* sp. | f | TS |
|  | *Coccoloba lucidula Benth.* | b | TS |
| Quiinaceae | *Lacunaria crenata (Tul.) A.C. Sm.* | f | TS |
|  | *Quiina* sp. | f | [41] |
| Rapateaceae | *Rapatea paludosa Aubl.* | b | TS |
| Rhamnaceae | *Ampelozizyphus amazonicus Ducke* | b | [43] |
| Rubiaceae | *Borojoa* sp. | f | [83] |
|  | *Carapichea guianense Aubl.* | b | TS |
|  | *Coussarea brevicaulis K. Krause* | b | [43] |
|  | *Diodis* sp. | b | [67] |
|  | *Duroia* sp. | f | [39] |
|  | *Faramea guianensis (Aubl.) Bremek.* | b | TS |
|  | *Faramea morilloi Steyerm.* | b | [23] |
|  | *Faramea multiflora A. Rich. ex DC.* | b | TS,[43] |
|  | *Genipa americana L.* | f | TS,[39,83] |
|  | *Genipa* sp. | f | TS |
|  | *Palicourea* spp. | f | [83] |
|  | *Psychotria capitata Ruiz & Pav.* | b | TS,[43] |
|  | *Psychotria cartagoensis Nepokroeff* | b | [43] |
|  | *Psychotria cincta Standl.* | b | [43] |
|  | *Psychotria cupularis (Müll. Arg.) Standl.* | b | TS |
|  | *Psychotria deflexa DC.* | b | [43] |
|  | *Psychotria iodotricha Müll. Arg.* | b | [43] |
|  | *Psychotria kappleri (Miq.) Müll. Arg. ex Benoist* | f | TS,[41] |
|  | *Psychotria loretensis Standl.* | b | [43] |
|  | *Psychotria lupulina Benth.* | b | [43] |
|  | *Psychotria mapourioides DC.* | b | TS |
|  | *Psychotria medusula Müll. Arg.* | b | TS |
|  | *Psychotria microbotrys Ruiz ex Standl.* | b | TS |
|  | *Psychotria poeppigiana Müll. Arg.* | b | [23,43] |
|  | *Psychotria racemosa (Aubl.) Raeusch.* | b | TS,[23] |
|  | *Psychotria tenuicaulis K. Krause* | b | [43] |
|  | *Psychotria* spp. | f | TS,[83,89] |
|  | *Rudgea guianensis (A. Rich.) Sandwith* | b | TS |
|  | *Warszewiczia coccinea (Vahl) Klotzsch* | b | [43] |
|  | indet. | f | TS,[25,39] |
| Rutaceae | *Citrus* sp. | f | [67] |
|  | indet. | b | TS |
| Sapindaceae | *Allophylus leucoclados Radlk.* | b | TS |
|  | *Paullinia rugosa Benth. ex Radlk.* | b | TS |
|  | *Serjania oblongifolia Radlk.* | b | TS |
|  | *Talisia* sp. | b | TS |
|  | *Talisia* sp. | f | [39] |
|  | indet. | f | [83] |
| Sapotaceae | *Chrysophyllum pomiferum (Eyma) T.D. Penn.* | f | TS |
|  | *Chrysophyllum sp.* | f | [41] |
|  | *Diploon cuspidatum (Hoehne) Cronquist* | f | TS |
|  | *Ecclinusa guianensis Eyma* | f | TS |
|  | indet. | b | [23] |
|  | indet. | f | TS,[23,25] |
|  | *Manilkara bidentata (A. DC.) A. Chev.* | f | TS,[41] |
|  | *Micropholis cf. egensis (A. DC.) Pierre* | f | [23] |
|  |  | b | [23,43] |
|  | *Micropholis melinoniana Pierre* | b | [23] |
|  |  | f | TS,[23] |
|  | *Micropholis* sp. | f | TS |
|  | *Pouteria egregia Sandwith* | f | TS |
|  | *Pouteria franciscana Baehni* | f | TS |
|  | *Pouteria guianensis Aubl.* | b | [43] |
|  | *Pouteria* spp. | f | TS,[43,83] |
|  | *Pradosia ptychandra (Eyma) T.D. Penn.* | f | TS |
|  | *Pradosia surinamensis* | f | [39] |
|  | *Sarcaulus* spp. | f | [83] |
| Simaroubaceae | *Simarouba amara Aubl.* | f | [83] |
| Siparunaceae | *Siparuna cristata or decipiens (Poepp. & Endl.) A. DC. / (Tul.) A. DC.* | f | TS |
| Smilacaceae | *Smilax campestris Griseb.* | b | [67] |
|  | *Smilax* sp. | b | TS |
| Solanaceae | *Cestrum microcalyx Francey* | b | [43] |
|  | *Cestrum* sp. | b | [43] |
|  | *Cestum* sp. | f | [83] |
|  | *Solanum* sp. | b | [43] |
|  | *indet.* | f | [89] |
| Sterculiaceae | *Guazuma ulmifolia Lam.* | f | [83] |
|  | *Sterculia pruriens (Aubl.) K. Schum.* | b | TS |
|  | *Theobroma cacao L.* | f | TS |
| Symplocaceae | *Symplocos martinicensis Jacq.* | b | TS |
| Tectariaceae | *Tectaria trifolia (Alderw.) C. Chr.* | b | TS |
| Thelypteridaceae | *Thelypteris glandulosa (Desv.) Proctor* | b | TS |
|  | *Thelypteris leprieuri (Hook.) R.M. Tryon* | b | TS |
|  | *Thelypteris macrophylla (Kunze) C.V. Morton* | b | TS |
| Thurniaceae | *Thurnia sphaerocephala (Rudge) Hook. f.* | b | TS |
| Ulmaceae | *Celtis iguanaea (Jacq.) Sarg.* | b | TS |
| Urticaceae | *Cecropia latiloba Aubl.* | b | [23] |
|  | *Cecropia obtusa Mart.* | f | TS |
|  | *Cecropia sciadophylla Mart.* | b | [23] |
|  |  | f | TS |
|  | *Cecropia* sp*.* | b | [43] |
|  | *Cecropia* spp. | f | [83] |
|  | *Pourouma bicolor Mart.* | b | [23] |
|  |  | f | [23] |
|  | *Pourouma guianensis Aubl.* | b | [23] |
|  |  | f | [23] |
|  | *Pourouma minor Benoist* | b | [23] |
|  |  | f | [83] |
|  | *Pourouma* sp. | f | TS,[43] |
| Verbenaceae | *Lantana camara L.* | b | [67] |
|  | *Vitex megapotamica (Spreng.) Moldenke* | b | [67] |
| Violaceae | *Amphirrox latifolia Mart.* | b | [23] |
|  | *Leonia glycycarpa Ruiz & Pav.* | b | [43] |
|  | *Leonia* sp. | b | [23] |
|  | *Rinorea flavescens (Aubl.) Kuntze* | b | [23] |
|  | *Rinorea riana Kuntze* | b | TS |
|  | *Rinorea* spp. | f | [83] |
|  | *Rinoreocarpus ulei (Melch.) Ducke* | f | [83] |
| Vochysiaceae | *Erisma* sp. | b | TS |
|  | *Vochysia lanceolata Stafleu* | b | [43] |
|  | *Vochysia lomatophylla Standl.* | b | [43] |
|  | *Vochysia* sp. | f | [41] |
| Zingiberaceae | *Renealmia monosperma* Miq. | b | TS |
|  |  |  |  |

TS stands for "This study"
